# Bibliographical

**Published:** 1891-01

**Authors:** 


### Bibliographical.

A Treatise on the Irregularities of the Teeth and Their Corrections, including with the author's practice other current methods. Illustrated with nearly 2,000 engravings (not embracing those in the third volume), by Jno. Nutling Farrar, M.D., D.D.S., graduate of Jefferson Medical College, Pa., and of Pa. College of Dental Surgery; member of New York County Medical Society; Medical Society of the County of Kings; International Medical Congress, IX; New York City District Dental Society of the State of New York; American Dental Association; Brooklyn Dental Society; honorary member of American Academy of Dental Science, and of Wisconsin State Dental Society; formerly lecturer in Pennsylvania College of Dental Surgery; member of the New York Academy of Science, and of the Metropolitan Museum of Fine Arts, New York.

Volume I,

Contains 730 pages. Some review of this work will be made in an early issue of the Register.

The Micro-Organisms of the Human Mouth, the Local and General Diseases Which Are Caused by Them, by Wiloughby D. Miller, D.D.S., M.D., Professor at the University of Berlin, with one hundred and twenty-eight illustrations, one chromo-lithographic, and two photo-micrographic plates. This work has recently come to hand and will be reviewed in a more extended notice in the next number of the Register.

